# *Discorpy*: algorithms and software for camera calibration and correction

**DOI:** 10.1107/S1600577525002267

**Published:** 2025-04-09

**Authors:** Nghia T. Vo

**Affiliations:** a National Synchrotron Light Source II Brookhaven National Laboratory Upton NY11973 USA; Tohoku University, Japan

**Keywords:** *Discorpy*, camera calibration, radial distortion, perspective distortion, optical center determination, tomography

## Abstract

Camera or lens-based detector calibration is essential for spatial accuracy in applications like dimensional tomography, optical metrology, and computer vision. Many methods and software exist yet there is still a lack of approaches that achieve both high accuracy and robustness while being easy to use and capable of handling a wide range of distortions. Radial lens distortion is common in high-resolution X-ray detector optics used in parallel-beam tomography at synchrotrons. Achieving sub-pixel accuracy requires calibrating with an optical target image. Although methods for characterizing radial distortion are well established, acquired images often also include perspective distortion and optical center offset. Here, we present our approaches to individually characterize and correct both types of distortion using a single calibration image, implemented in the *Discorpy* software.

## Abstract 2

*Discorpy*, a Python package for camera calibration, provides robust tools to independently correct radial and perspective distortion of varying strengths using a single calibration image.

## 1. Introduction

**Figure 1:**
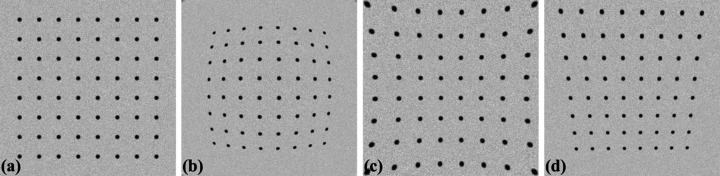
(*a*) Undistorted image. (*b*) Barrel distortion. (*c*) Pincushion distortion. (*d*) Perspective distortion.

In lens-coupled detectors or cameras, two major types of distortion commonly observed in acquired images are radial and perspective distortions (Fig. 1 ▸). Radial distortion occurs when the magnification of a lens varies with the radius from the optical axis, resulting in barrel distortion or pincushion distortion [Figs. 1 ▸(*b*) and 1(*c*)]. Perspective distortion arises when either the lens plane is not parallel to the camera sensor plane, known as tangential distortion [Fig. 1 ▸(*d*)], or the object plane is not parallel to the camera sensor plane; or a combination of both.

For correcting radial and/or perspective distortions, it is essential to have a model that maps between distorted and undistorted spaces. Mapping from undistorted to distorted space is known as forward mapping, while the reverse process is referred to as backward or inverse mapping. Numerous models are available in the literature (Brown, 1971 ▸; Clarke & Fryer, 1998 ▸; Ricolfe-Viala & Sanchez-Salmeron, 2010 ▸), such as polynomial, logarithmic, field-of-view, or matrix-based models, which describe the relationship between undistorted and distorted spaces. Some models are designed specifically for one type of distortion, whereas others address both types and include the location of the optical center. Once a model is selected, a practical approach can be developed to calculate the parameters of this model.

Among many models for characterizing radial distortion, the polynomial model is versatile enough to correct both small-to-medium distortion with sub-pixel accuracy (Haneishi *et al.*, 1995 ▸; Vo *et al.*, 2015 ▸) and strong distortion such as fisheye effects (Basu & Licardie, 1995 ▸). For a comprehensive overview of radial distortion correction methods, readers are recommended to refer to the review articles by Hughes *et al.* (2008 ▸, 2010*a* ▸) and Ricolfe-Viala & Sanchez-Salmeron (2010 ▸). When perspective distortion and optical center offset are present, radial distortion can be calibrated using two main approaches. One approach is iterative optimization, which uses a cost function to ensure that corrected lines in the undistorted image appear straight (Devernay & Faugeras, 2001 ▸). Although it requires only a single calibration image, this method is computationally expensive and does not always guarantee convergence. The other approach relies on multiple calibration images to estimate all distortion parameters, as implemented in *OpenCV*. However, its accuracy is limited because the polynomial model uses only even-order terms (Zhang, 2002 ▸). Therefore, a method for calibrating radial distortion in the presence of other distortions that achieves high accuracy using only a single calibration image is both practical and crucially needed.

For synchrotron applications, particularly at beamline I12 (Drakopoulos *et al.*, 2015 ▸) of Diamond Light Source (DLS), UK, practical methods for calculating coefficients of the polynomial model to achieve sub-pixel accuracy, critical for X-ray parallel-beam tomography, have been developed and well documented by Vo *et al.* (2015 ▸). These methods focus only on correcting radial distortion, as the optical design of I12’s detection system eliminates perspective distortion. At the same facility, beamline I13 (Rau *et al.*, 2011 ▸), which offers higher resolution tomography than I12, is more challenging in aligning the distortion target to remove perspective distortion due to its optical design. In such cases, developing approaches that allow independent characterization of both distortion types using a single calibration image is essential. Here, we present our methods to achieve this and their implementation in the *Discorpy* software. Additionally, pre-processing algorithms have been developed to extract reference points from dot-pattern, line-pattern, and chessboard images; robustly group reference points line-by-line; and detect lines even when strongly curved. Methods for calculating the distortion center in the presence of significant perspective distortion have also been introduced to streamline the data processing workflow. The paper is structured as follows. The *Methods*[Sec sec2] section outlines the step-by-step workflow for calibrating radial distortion using a calibration image. The *Results*[Sec sec3] section demonstrates the application of distortion calibration and correction at tomographic beamlines at DLS. Finally, the *Conclusion*[Sec sec4] summarizes the key findings

## 2. Methods

**Figure 2:**
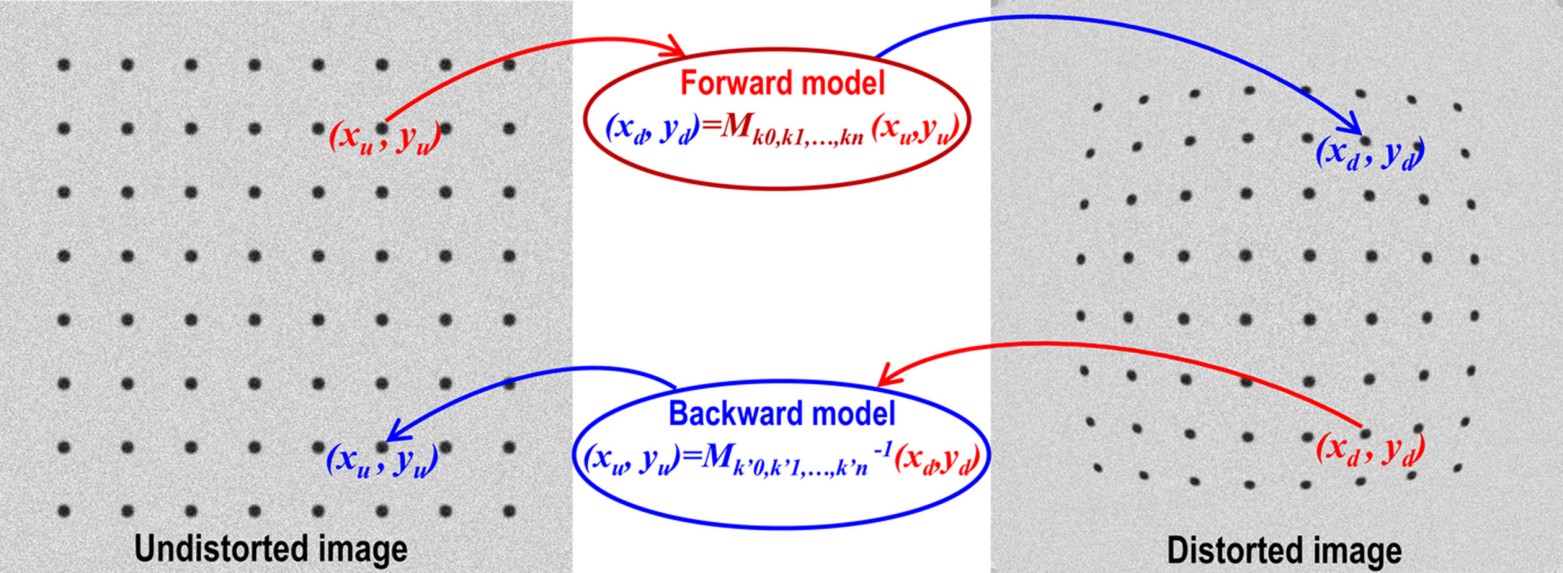
Demonstration of the forward mapping and backward mapping between distorted and undistorted spaces.

The purpose of a calibration process is to find parameters of a model that maps between distorted and undistorted spaces (Fig. 2 ▸). To calculate these parameters, we must determine the coordinates of reference points in the distorted space and their corresponding positions in the undistorted space. Reference points can be extracted using a calibration image, which may be a dot pattern, line pattern, or chessboard (checkerboard). For example, the center of mass of a dot pattern, the intersection points of a line pattern, or the corners of a chessboard image can serve as reference points. By ensuring that lines formed by these points are straight, equidistant, parallel, or perpendicular, we can estimate the locations of these reference points in the undistorted space with high accuracy. The following sections present the calibration process step by step, from extracting reference points to calculating model parameters and correcting the image.

### 2.1. Extracting reference points from a calibration image

**Figure 3:**
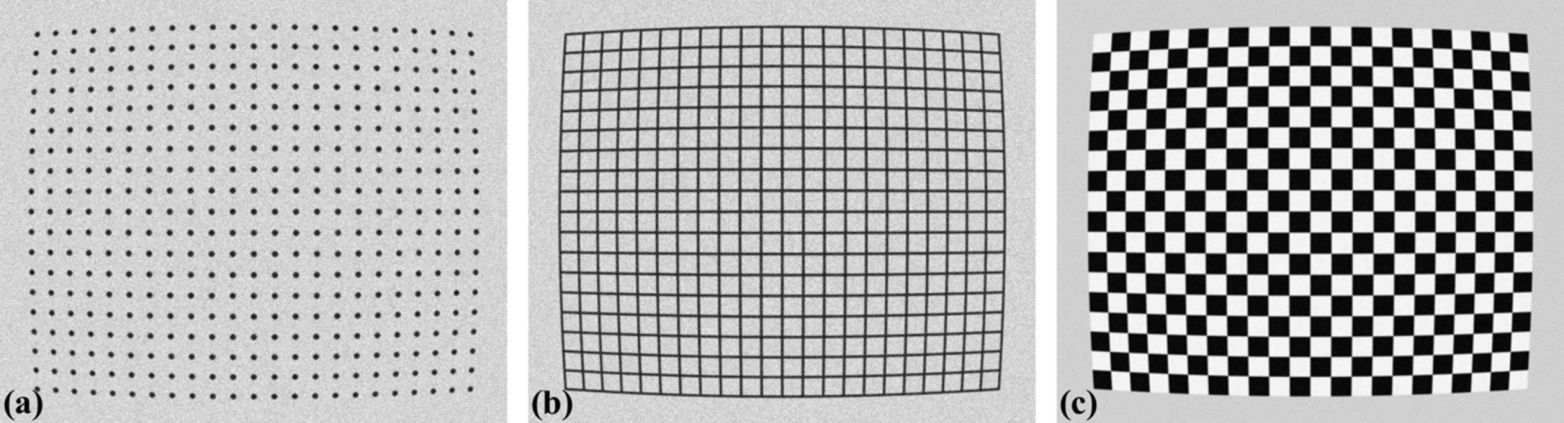
Common types of calibration images: (*a*) dot-pattern image, (*b*) line-pattern image, (*c*) chessboard image.

A calibration image provides reference points that can be extracted using various image processing techniques. As shown in Fig. 3 ▸, several types of calibration images are commonly used. A dot-pattern image is the simplest to process, requiring only dot segmentation and center-of-mass calculation for each dot. A line-pattern image requires line detection techniques to identify reference points along the detected lines or at their intersections. For a chessboard image, corner detection methods can be applied, or a gradient filter can be used to convert it into a line-pattern image, followed by line detection. Different applications use different types of distortion targets. For example, NIST-standard dot patterns, available from commercial suppliers, are used to characterize detector systems in micro-tomography. For sub-micrometer systems, line patterns are easier to fabricate. Chessboard patterns, on the other hand, are widely used in computer vision applications

Good practices for acquiring high-quality calibration images include maximizing the grid pattern’s coverage within the camera’s field of view, minimizing background light variance, ensuring the pattern object is flat, and reducing perspective effects by adjusting the target or camera to be as parallel as possible. While *Discorpy* can handle challenging images, following these practices helps ensure the calibration workflow runs smoothly with minimal need for adjusting method parameters.

However, even with these precautions, the quality of acquired images may still vary, presenting challenges such as non-uniform backgrounds or additional features beyond the intended grid pattern. These variations can impact the performance of subsequent processing methods in the calibration workflow. To address these challenges, preprocessing methods have been developed in *Discorpy* to handle variations in both image quality and calibration image types.

#### 2.1.1. Preprocessing methods for a dot-pattern image

To segment dots from an image, we must ensure that the background intensity is uniform. This can be achieved through a normalization step where the background is extracted using two approaches: applying a strong Gaussian-based low-pass filter; or using a median filter with a large-sized window (*i.e.* larger than the dot size). After the background is extracted, normalization is performed by dividing the original image by the background image. For binarization, the methods used are Otsu’s method (Otsu, 1979 ▸) or the sorting-based algorithm described by Vo *et al.* (2018 ▸).

**Figure 4:**
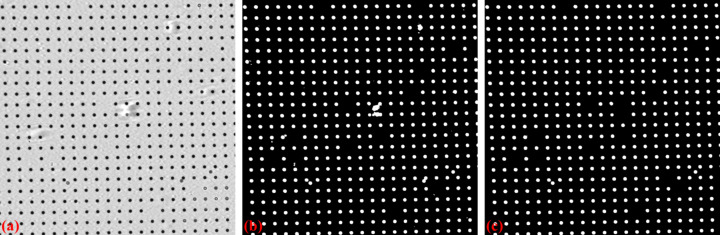
Demonstration of removing non-dot objects: (*a*) dot-pattern image (X-ray target), (*b*) binary image, (*c*) image with non-dot objects removed.

For a custom-made X-ray dot target, non-dot features may be introduced by the optics system or defects on the scintillator. To address these issues, *Discorpy* provides two methods to distinguish whether a binary object is a valid dot or not. In the first approach, the median size of the dots is determined, and only objects with sizes close to this median are retained (Fig. 4 ▸). In the second approach, the ratio between the largest and smallest axes of the best-fit ellipse is calculated. Objects with ratios outside a specified range are removed.

**Figure 5:**
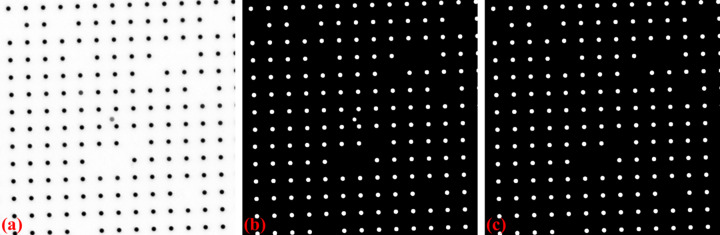
Demonstration of removing misplaced dots: (*a*) image with a misplaced dot, (*b*) binary image, (*c*) image with misplaced dot removed.

Custom-made dot-patterns may have dots placed in incorrect positions, as shown in Fig. 5 ▸. In *Discorpy*, a misplaced dot is identified by comparing its distances with the four nearest dots. If none of these distances is close to the median distance between the two nearest dots, the dot is removed. However, this method should not be used for images with strong distortion, where the distance between two nearest dots can vary significantly depending on their proximity to the optical center. A more generic approach to address this issue is introduced in the next step of the processing pipeline.

#### 2.1.2. Preprocessing methods for a line-pattern image and a chessboard image

**Figure 6:**
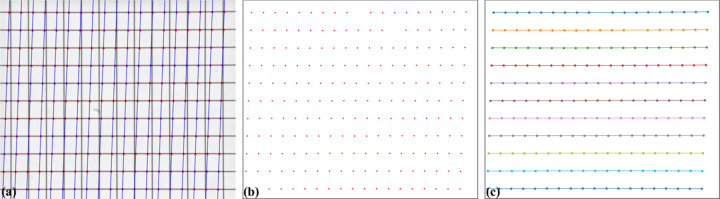
Proposed detecting line method: (*a*) multiple crossing-lines are used to locate extrema points, (*b*) points extracted, (*c*) points after grouped into lines.

As lines in the image appear curved due to radial distortion, using well known line-detection methods like Hough transform-based approaches (Duda & Hart, 1972 ▸) is not always feasible. We introduce a simple method for detecting curved lines, where points on each line are identified by locating local extrema on an intensity profile generated by plotting across the image in a closely perpendicular direction (Fig. 6 ▸). Multiple line profiles are generated to detect points on lines at various locations, and these points are then grouped by line in the next step.

Behind the scenes, the method for locating points involves detecting local minimum or maximum points from an intensity line profile. To improve the robustness of this approach, a technique to verify whether a local minimum or maximum is a valid point has been added to *Discorpy*. This involves Gaussian-based fitting and a quality judgment parameter.

For chessboard images, there are two approaches to extract reference points. In the first approach, the image is converted to a line-pattern image by applying a gradient filter, and then methods relevant to this type of pattern are used to obtain reference points. In the second approach, the edges of the boundaries between neighboring chessboard squares are located by generating an intensity line profile similar to the previous step. A sliding linear fit window is then applied along this line to transform edge detection into a peak detection problem; following this, the method of locating local minimum or maximum points, as described above, is used.

#### 2.1.3. Supporting methods

To enhance the robustness and automation of preprocessing workflows, several supporting methods have been integrated into the software. These methods are for various tasks, such as calculating the sizes and distances of dots and lines, determining the slopes of horizontal and vertical lines, and applying rotation transformation to point coordinates. Additional capabilities include masking out image features or points that fall outside a parabolic mask and selectively removing subsets of points.

### 2.2. Grouping reference-points into horizontal lines and vertical lines

**Figure 7:**
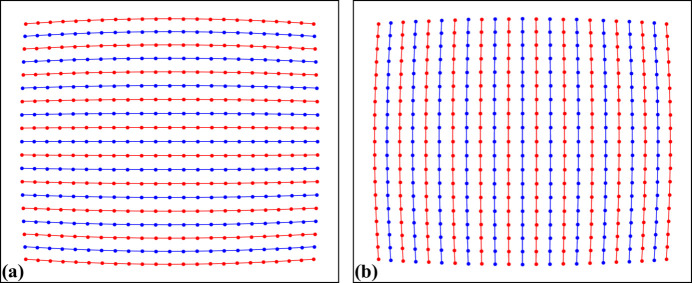
(*a*) Points are grouped into horizontal lines. (*b*) Points are grouped into vertical lines.

Different techniques for calculating distortion model parameters use reference points in various ways. *Discorpy* developed methods that groups reference points into horizontal and vertical lines (Fig. 7 ▸), represents them using parabolic fit coefficients (Bailey, 2002 ▸; Vo *et al.*, 2015 ▸), and then uses these coefficients to calculate distortion parameters

The previous steps generate a list of independent points, which may either belong to a line or be outliers. In the current step, these points must be grouped by line, separately in horizontal and vertical directions. The grouping step is crucial in the data processing workflow as it significantly influences the performance of subsequent methods. In *Discorpy*, the grouping method involves searching the neighbors of a point to determine whether they belong to the same line. This search is guided by the slope of the grid, the nominal distance between lines, a tolerance parameter, and an allowable number of missing points. Depending on the quality of the calibration image, users may need to adjust the parameters of preprocessing methods and the grouping method to achieve optimal results.

**Figure 8:**
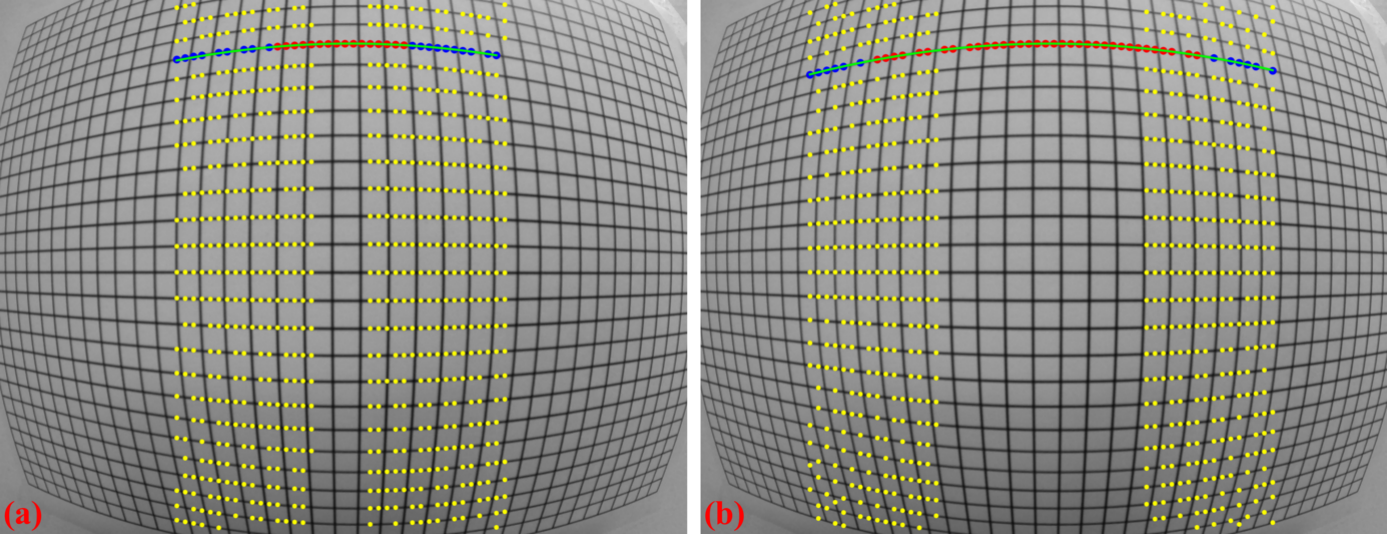
Demonstration of the grouping method for strongly curved lines: (*a*) initial search window, (*b*) next search window.

The introduced method works well for small-to-medium distortions where lines are not strongly curved. However, for fisheye images, where lines are significantly curved, using the slope (orientation) of the grid to guide the search is not effective. To address this problem, a different approach is used, where points are grouped from the middle of the image outward, guided by a polynomial fit to locate nearby points. The process includes two steps: first, in the horizontal direction, points around the center of the image, where distortion is minimal, are grouped line by line using the previous method. For each group of points, a parabolic fit is applied. The search window then moves to the next slab (left and right from the center) and selects points close to the fitted parabola. The parabolic fit is then updated to include the newly grouped points. This search window continues moving to both sides until the entire image is covered (Fig. 8 ▸).

After points are grouped line-by-line, the coordinates of points on each group are fitted to parabolas in which horizontal lines are represented by 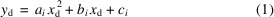
and vertical lines by 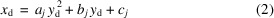
where *i* and *j* are the indices of the horizontal and vertical lines, respectively. Note that the origin of *x*_d_ and *y*_d_ corresponds to the image coordinate system, *i.e.* the top-left corner of the image. The subscript ‘d’ is to indicate that points are in the distorted space. As mentioned in Section 2.1.1, misplaced dots or outliers can be removed after grouping. Points in the same group are fitted with a parabolic curve, and any points with a distance larger than a specified threshold (in pixel units) from their fitted positions are removed.

### 2.3. Calculating the center of radial distortion

For methods of calculating coefficients of the polynomial model of radial distortion, determining the distortion center is crucial and an independent step. If there is no perspective distortion, the center can be calculated by locating the intersection of two lines: the first line is formed using a slope and an intercept calculated by averaging the *b* coefficients and *c* coefficients of two horizontal parabolas, where the second-order coefficient *a* changes sign; the second line is formed similarly but using vertical parabolas. This center can be further refined using the routine described by Vo *et al.* (2015 ▸).

**Figure 9:**
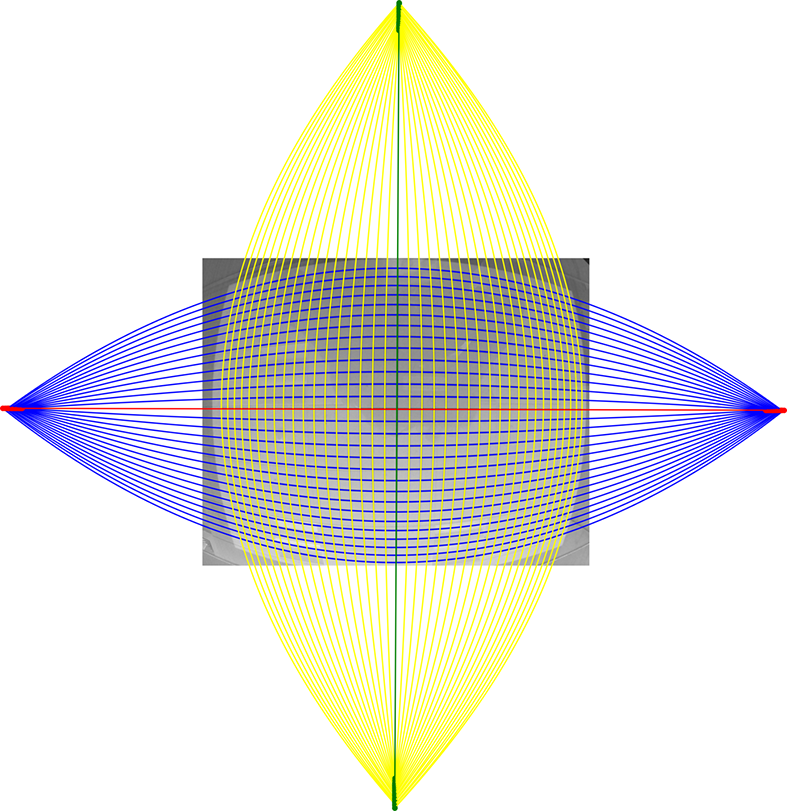
Demonstration of the proposed method for finding the distortion center.

For cases where perspective distortion is present and radial distortion is not strong, methods proposed by Bailey (2002 ▸) can be used. However, for strong radial distortion, we propose two approaches. These methods are based on the idea of using vanishing points (Hughes *et al.*, 2010*b* ▸) formed by the intersections of distorted line. However, unlike the approach described in that article, the method proposed in this paper uses parabola fitting instead of circular fitting, and it does not attempt to locate four vanishing points. Instead, the first approach involves finding the intersection points between parabolas with opposite signs of the *a*-coefficient at the same orientation, *e.g.* horizontal parabolas. A linear fit is then applied to these points. The same process is repeated for vertical parabolas. The intersection of the two fitted lines provides the distortion center (Fig. 9 ▸). This method works well for barrel radial distortion.

For broader applicability, the second approach is as follows. For each orientation, *e.g.* horizontal direction, the parabola with the absolute minimum *a*-coefficient is identified, then the intersection points between this parabola and the rest are calculated. A linear fit is applied to these points. The same routine is repeated for vertical direction parabolas. The intersection points of these two fitted lines determine the calculated center. This entire routine is repeated two or three times to refine the calculated center further by combining it with perspective distortion correction, as will be shown in the next section.

### 2.4. Correcting perspective distortion

**Figure 10:**
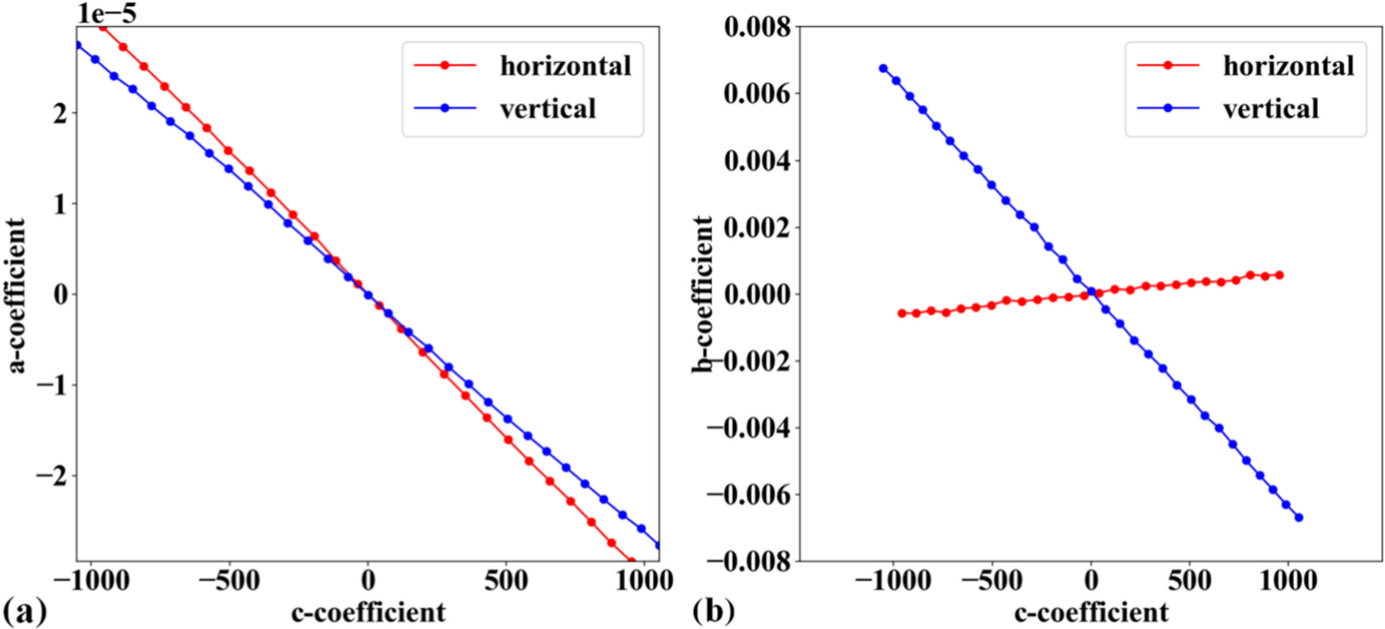
Effects of perspective distortion on parabolic coefficients: (*a*) relationship between *a*- and *c*-coefficients, (*b*) relationship between *b*- and *c*-coefficients. Note that the sign of the *b*-coefficients for vertical lines has been reversed to match the sign of the horizontal lines.

In practice, the target object used for acquiring a calibration image is often not aligned parallel to the sensor plane. This causes perspective distortion in the acquired image, which affects the accuracy of the calculated model for radial distortion. Perspective distortion can be detected by analyzing the parabolic coefficients of lines (Bailey, 2002 ▸), where the origin of the coordinate system is shifted to the distortion center before performing the parabola fitting. Fig. 10 ▸(*a*) shows a plot of *a*-coefficients against *c*-coefficients for horizontal lines [equation (1)] and vertical lines [equation (2)]. If perspective distortion is present, the slopes of the straight lines fitted to the plotted data are different. Another consequence is that *b*-coefficients vary with *c*-coefficients instead of remaining close to constant, as shown in Fig. 10 ▸(*b*).

**Figure 11:**
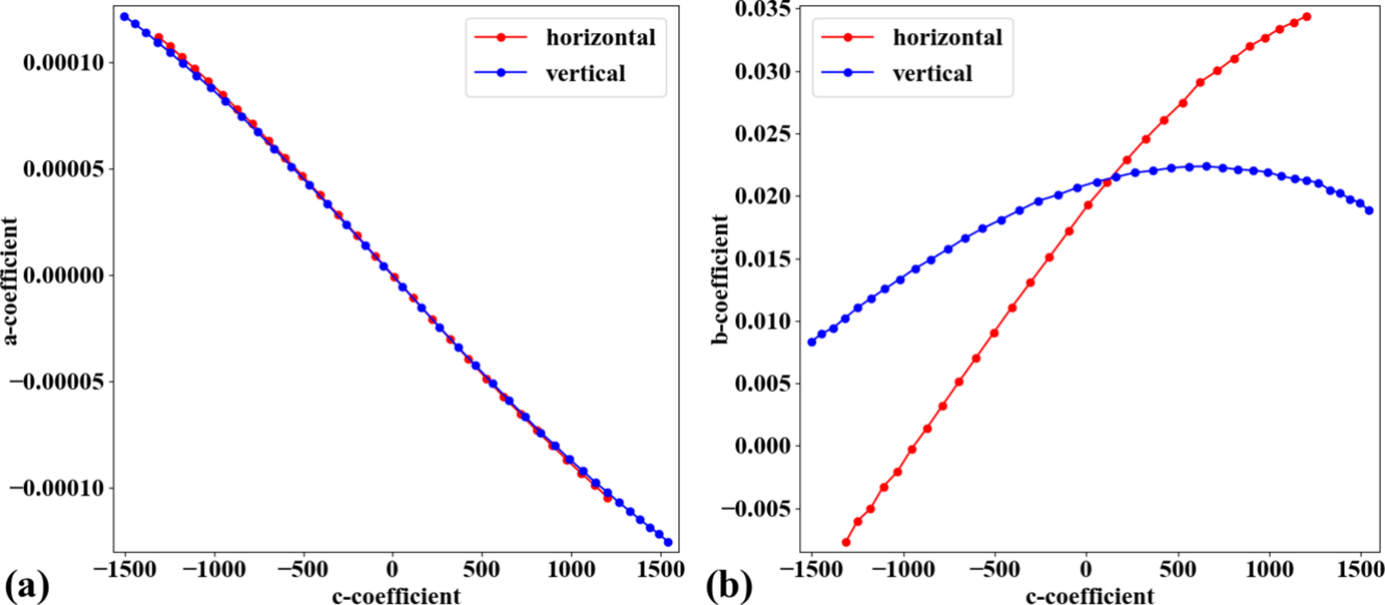
Effects of perspective distortion on parabolic coefficients of a strongly distorted grid: (*a*) relationship between *a*- and *c*-coefficients, (*b*) relationship between *b*- and *c*-coefficients. Note that the sign of the *b*-coefficients for vertical lines has been reversed to match the sign of the horizontal lines.

To calibrate radial distortion, perspective distortion must be corrected first. This can be achieved in two ways. In the first approach, the coefficients of the parabolic fit are adjusted. Specifically, the *a*- and *c*-coefficients of parabolas in one direction (*e.g.* horizontal) are corrected using a ratio calculated as the division between the average of the differences of *c*-coefficients in the other direction (vertical) and those in the same direction (horizontal). The *b*-coefficient is simply taken as the value at the intersection of the fitted lines, as shown in Fig. 10 ▸. This approach works well for small-to-medium radial distortion. However, for strong distortion, the graphs of *a*-versus-*c* coefficients and *b*-versus-*c* coefficients are not straight, as shown in Fig. 11 ▸. To address this problem, perspective distortion is calibrated using the fact that the representative lines between directions are perpendicular. Using the perspective model described by Criminisi *et al.* (1999 ▸), 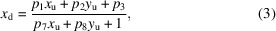

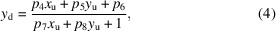
to determine the eight coefficients in equations (3) and (4) we need the coordinates of at least four pairs of distorted and undistorted points.

The following describes the second approach which calculates the coefficients of the above model and applies correction. First, the distortion center is determined using the vanishing points approach described in Section 2.3. The coordinates of the reference points are updated by shifting the origin from the top-left corner of the image to the distortion center, and the parabola fit is recalculated using the updated coordinates. Next, the intersection of four lines is found: two in the horizontal direction and two in the vertical direction, as follows. The first horizontal line is determined by averaging the *b*-coefficients and *c*-coefficients of parabolas with positive *a*-coefficients, while the second horizontal line is determined using the same approach but applied only to parabolas with negative *a*-coefficients. The two vertical lines are determined in a similar manner.

**Figure 12:**
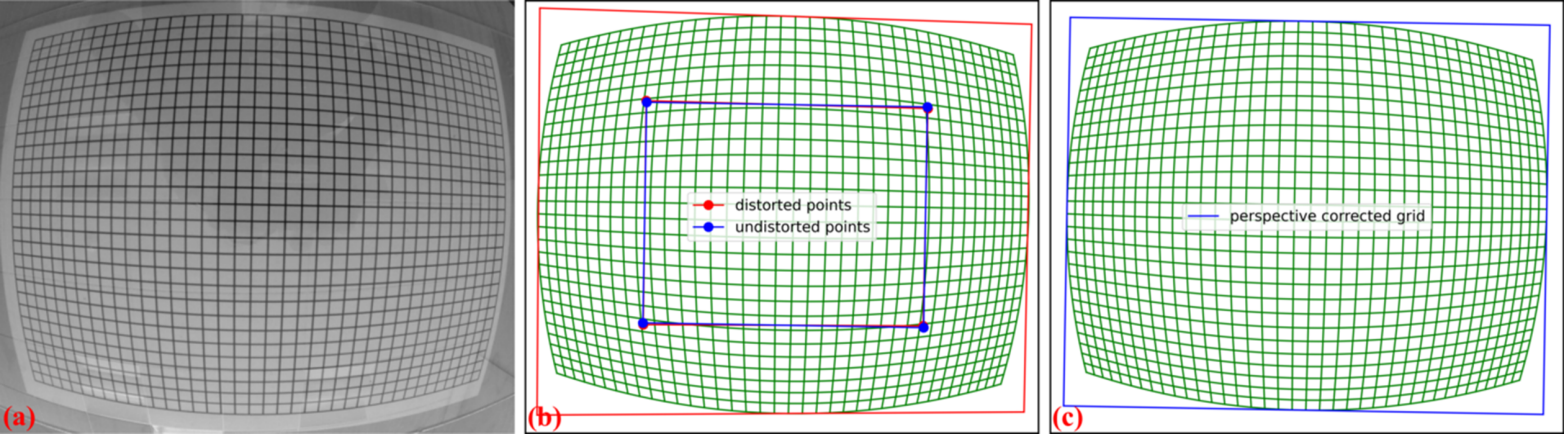
Demonstration of the method for characterizing perspective distortion: (*a*) line-pattern calibration image, (*b*) grid showing four distorted points and four undistorted points, (*c*) corrected grid. Note that the red outline frame in (*b*) and the blue outline frame in (*c*) highlight the perspective effect before and after correction.

From the four intersection points of these lines, the undistorted points can be calculated using the condition that the lines must be parallel in one direction and perpendicular in the other. The idea involves averaging the slopes and intercepts to determine the slopes and intercepts of these lines in the undistorted space, and then finding the intersection of the lines [Fig. 12 ▸(*b*)]. As the scale between the two spaces is uncertain, the average distance between points is used to define the scale. If the pixel size of the camera is known and the distance between lines is measured, the scale can be determined more accurately. In such cases, *Discorpy* provides an option to specify the scale of the undistorted space. Using these pairs of points between the distorted and undistorted spaces, perspective distortion coefficients are calculated from equations (3) and (4). The correction is then applied to all reference points and the results are used in the next step of calibration.

### 2.5. Calculating coefficients of polynomial models for radial distortion correction

The polynomial model is chosen as it is versatile enough to correct both small-to-medium distortion with sub-pixel accuracy (Vo *et al.*, 2015 ▸) and strong distortion such as fisheye effects (Basu & Licardie, 1995 ▸). The backward model is given by
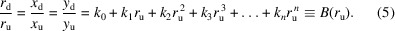
Here, (*x*_u_, *y*_u_) are the coordinates of a point in the undistorted space, and *r*_u_ is its distance from the distortion center. (*x*_d_, *y*_d_) and *r*_d_ represent the coordinates of a point and its distance from the center in the distorted space.

To calculate the coefficients of these models, it is necessary to determine the coordinates of reference points in both the distorted and undistorted spaces and solve a system of linear equations. As presented by Vo *et al.* (2015 ▸), the author simplifies this task by finding the intercepts, 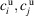, of undistorted lines instead. Using the assumption that lines are equidistant, 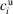 and 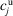 are calculated by extrapolating from a few lines near the distortion center as 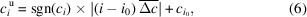

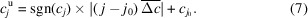
Here, the sgn(…) function returns the value of −1, 0, or 1 depending on whether its input is negative, zero, or positive. *i*_0_, *j*_0_ are the indices of the horizontal and vertical line closest to the distortion center. 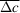 is the average of the difference of *c*_*i*_ values near the distortion center. 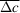 can be refined further by varying it around an initial guess and minimizing the cost function 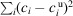, as provided in the software.

In practice, it may be necessary to calculate the coefficients of a backward model given the coefficients of the corresponding forward model, or vice versa. This process is straightforward: a list of reference points can be generated, and their positions in the opposite space can be calculated using the known model. From the data points in both spaces, a system of linear equations can be formulated and solved to determine the coefficients of the opposite model. This functionality is available in *Discorpy*.

### 2.6. Correcting distorted images

**Figure 13:**
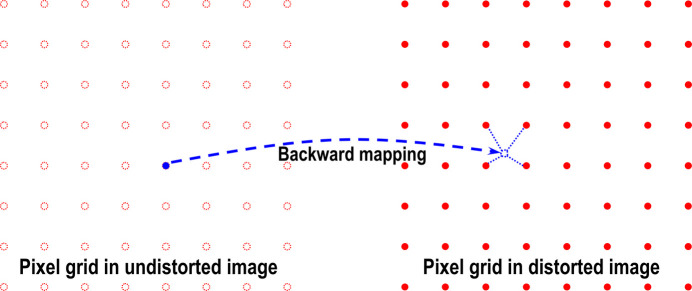
Demonstration of the backward mapping routine.

To correct distorted images, backward models are used because the values of pixels adjacent to a mapped point are known (Fig. 13 ▸). This simplifies the interpolation process. It is important to note that in the field of image processing the coordinate of a pixel conventionally refers to the top-left corner of an image. The calculated center of distortion is referenced to this origin.

For radial distortion, given (*x*_u_, *y*_u_), which corresponds to the pixel indices (*j*, *i*) in the column and row, of a pixel in the undistorted image, distortion center (*x*_c_, *y*_c_), and the coefficients (*k*_0_, *k*_1_,…, *k*_*n*_) of a backward model, the correction routine is as follows:

(i) Translate the coordinates: *x*_u_ = *x*_u_ − *x*_c_; *y*_u_ = *y*_u_ − *y*_c_.

(ii) Calculate: 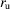 = 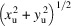; 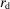 = *r*_u_(*k*_0_ + *k*_1_*r*_u_ + 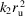 + 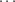 + 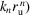.

(iii) Calculate: *x*_d_ = *x*_u_*r*_d_/*r*_u_; *y*_d_ = *y*_u_*r*_d_/*r*_u_.

(iv) Translate the coordinates: *x*_d_ = *x*_d_ + *x*_c_; *y*_d_ = *y*_d_ + *y*_c_.

(v) Find four nearest pixels in the distorted image by combing the sets [floor(*x*_d_), ceil(*x*_d_)] and [floor(*y*_d_), ceil(*y*_d_)]. Clip values that are outside of the image size.

(vi) Interpolate the value at (*x*_d_, *y*_d_) using the values of four nearest pixels and assign the result to the corresponding point in the undistorted image.

Correcting perspective distortion is straightforward. Given (*x*_u_, *y*_u_) and perspective coefficients, equations (3) and (4) are used to calculate (*x*_d_, *y*_d_). Then, the image value at this location is calculated by interpolation, as explained above. It is important to note that the scaling, translation, and rotation of reference points can be controlled to achieve the desired corrected image. For instance, the real distance between lines can be ensured given the pixel size, which is crucial for metrology applications.

In addition to image distortion correction, *Discorpy* provides utility methods for result evaluation, such as:

(i) Unwarping (undistorting) points using forward and backward models.

(ii) Evaluating the straightness of a line of points by calculating their distances to the fitted line.

(iii) Unwarping slices of a stack of images, which is particularly useful for tomographic data.

### 2.7. Software structure

Based on the workflow described above, *Discorpy* is designed with four basic modules:

(i) Loader-saver module: used to load images and save outputs, such as images, metadata files, or plots.

(ii) Pre-processing module: for extracting reference points from a calibration image and grouping points into lines.

(iii) Processing module: for calculating distortion parameters.

(iv) Post-processing module: for unwarping distorted lines, images, or slices and evaluating the accuracy of the correction results.

Additionally, the utility module provides methods such as generating simulated grid patterns, finding corresponding points between spaces, and supporting other operations for calibration workflows.

## 3. Results and applications

### 3.1. Calibrating radial distortion in the presence of perspective distortion and offset optical center

The key contribution of this work is the ability to calibrate radial distortion even in the presence of other distortions using a single calibration image, with the proposed methods performing well across a wide range of distortion strengths. This extends the applicability of the techniques distributed in *Discorpy*, making them practical for various use cases. The following presents calibration results of target images with different distortion strength.

**Figure 14:**
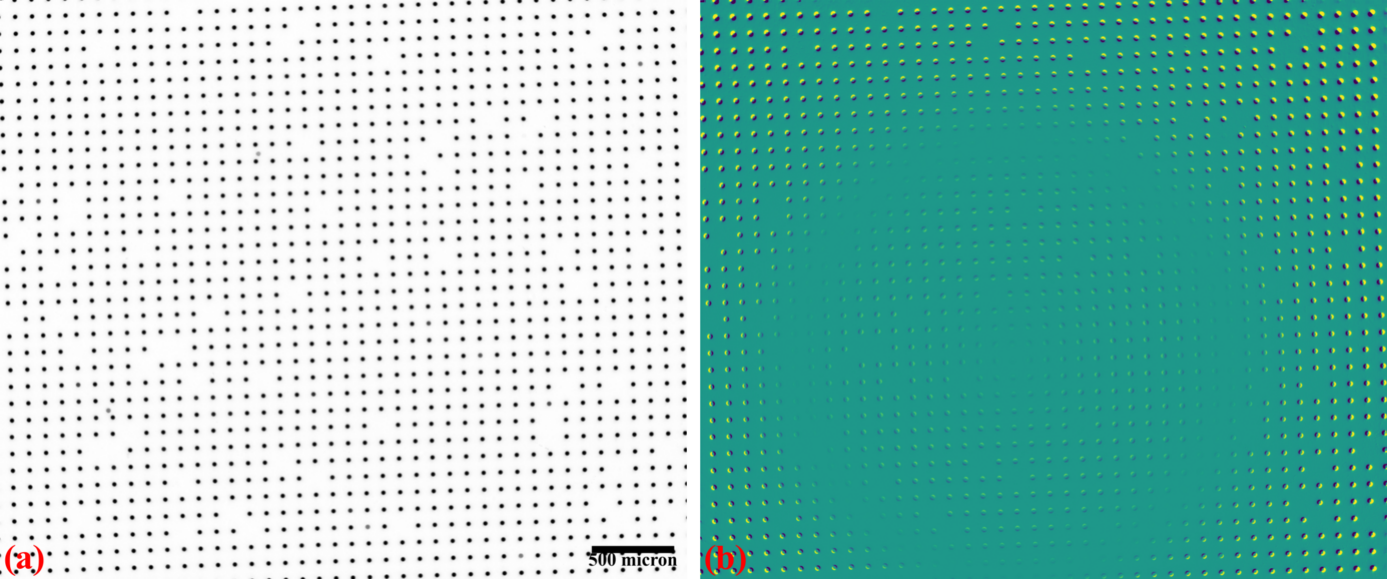
(*a*) X-ray dot-target image, (*b*) difference between the images before and after distortion correction.

**Figure 15:**
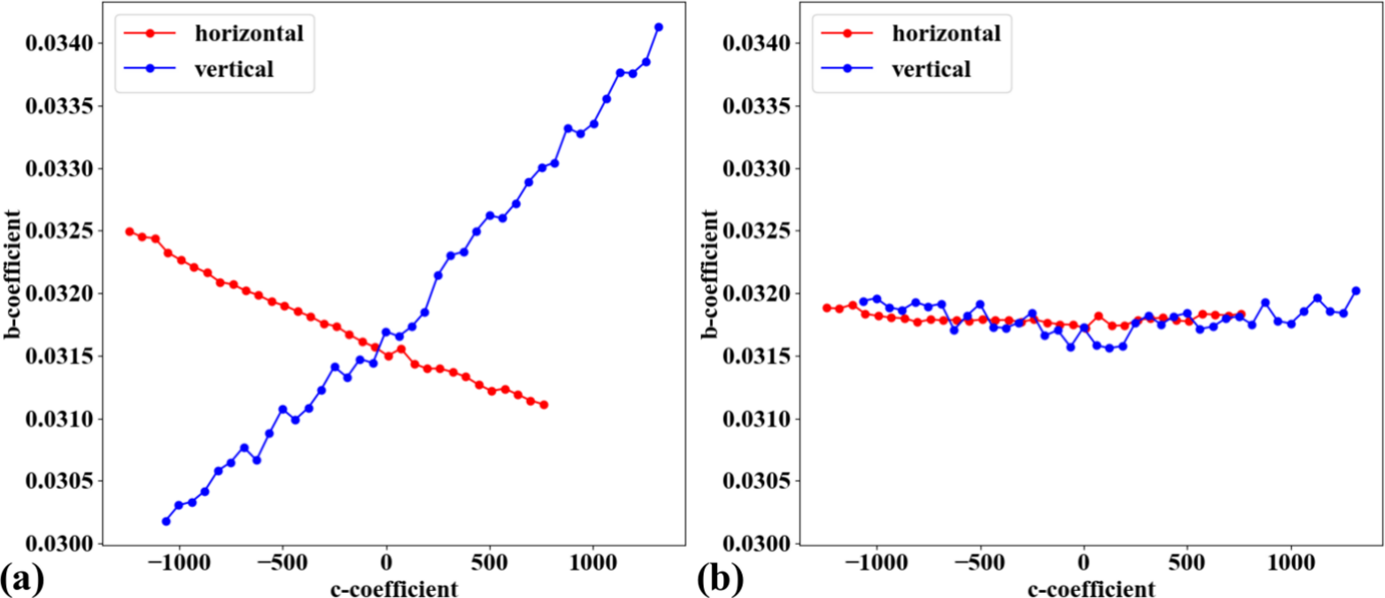
Relationship between *b*- and *c*-coefficients: (*a*) before perspective correction, (*b*) after perspective correction.

**Figure 16:**
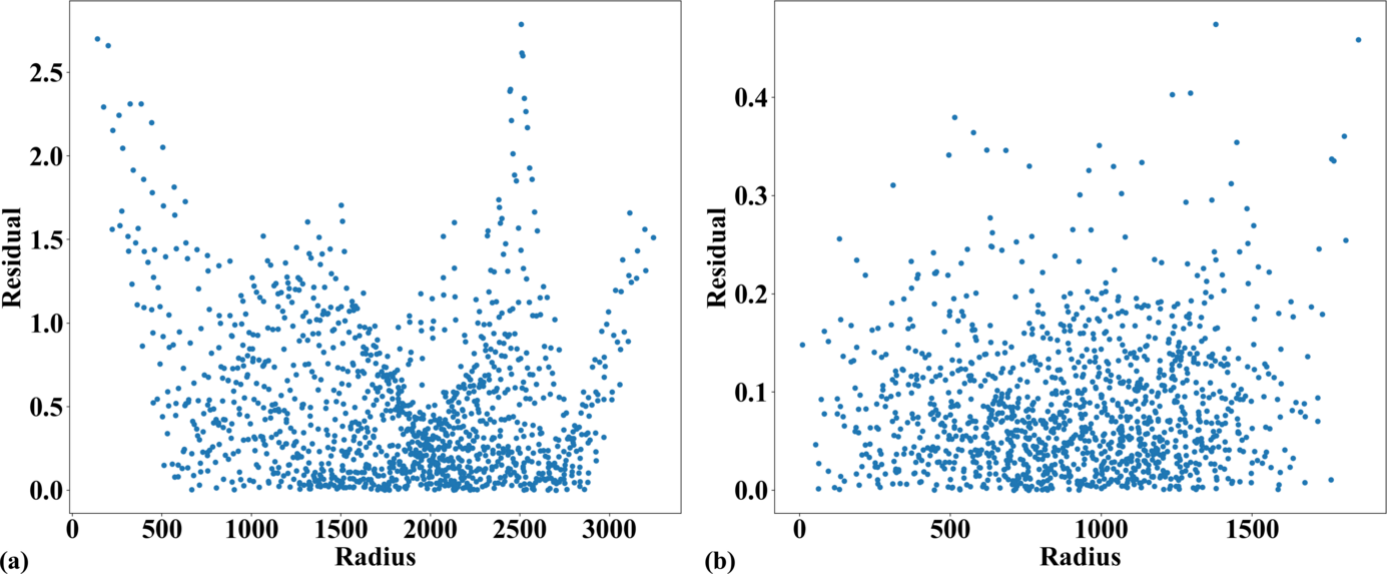
Plot of dot-centroid distances from their fitted straight line (horizontal direction) versus their distances from the origin: (*a*) before radial distortion correction, (*b*) after correction.

Fig. 14 ▸ shows an X-ray image of the dot target acquired at beamline I13 (1.63 µm pixel size, PCO Edge camera, 2560 × 2160 pixels). Analyzing the parabolic coefficients of the horizontal and vertical lines reveals the presence of perspective distortion, as shown in Fig. 15 ▸(*a*). Using the approaches described in previous sections, the distortion center is first calculated, followed by perspective distortion correction, as demonstrated in Fig. 15 ▸(*b*). With the perspective-corrected coordinates, the radial distortion coefficients are then computed. We evaluate the accuracy of the correction by measuring the distance of reference points from the fitted lines within their groups. Before radial distortion correction, the maximum deviation is around 2.5 pixels, which is reduced to below 0.5 pixels after correction (Fig. 16 ▸). This level of accuracy is crucial for tomography data, as will be demonstrated in the next section.

**Figure 17:**
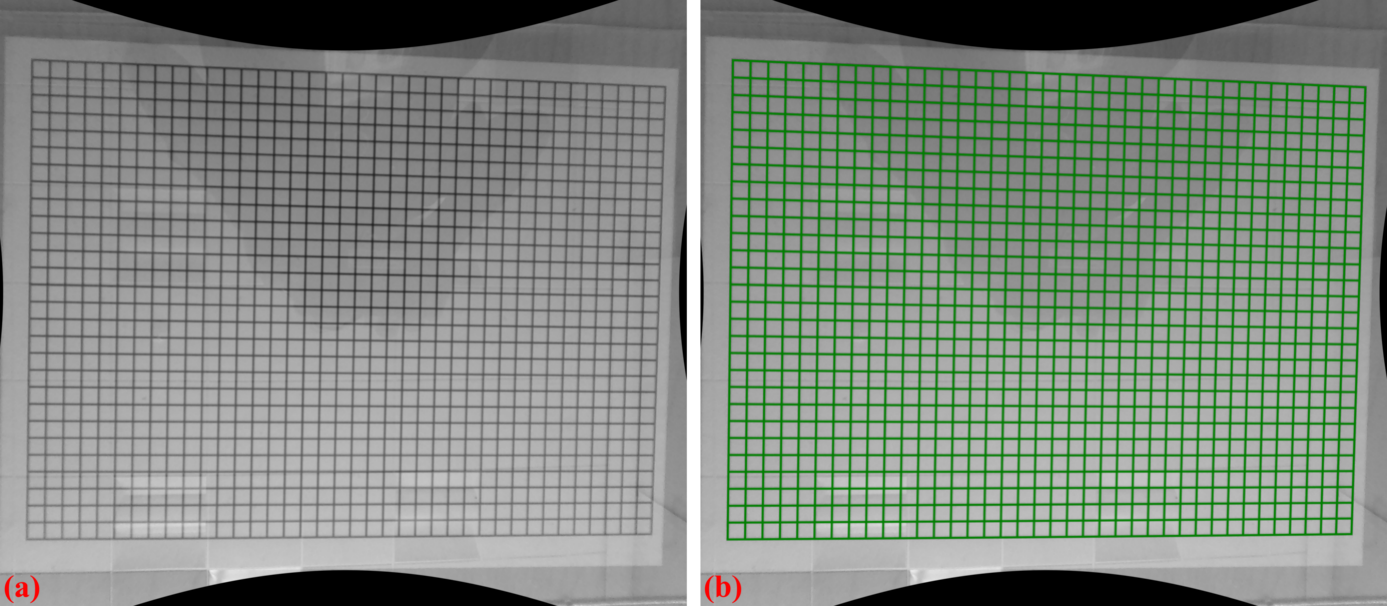
(*a*) Radial distortion correction of the image in Fig. 12 ▸(*a*); (*b*) radial distortion correction of the grid in Fig. 12 ▸(*b*).

**Figure 18:**
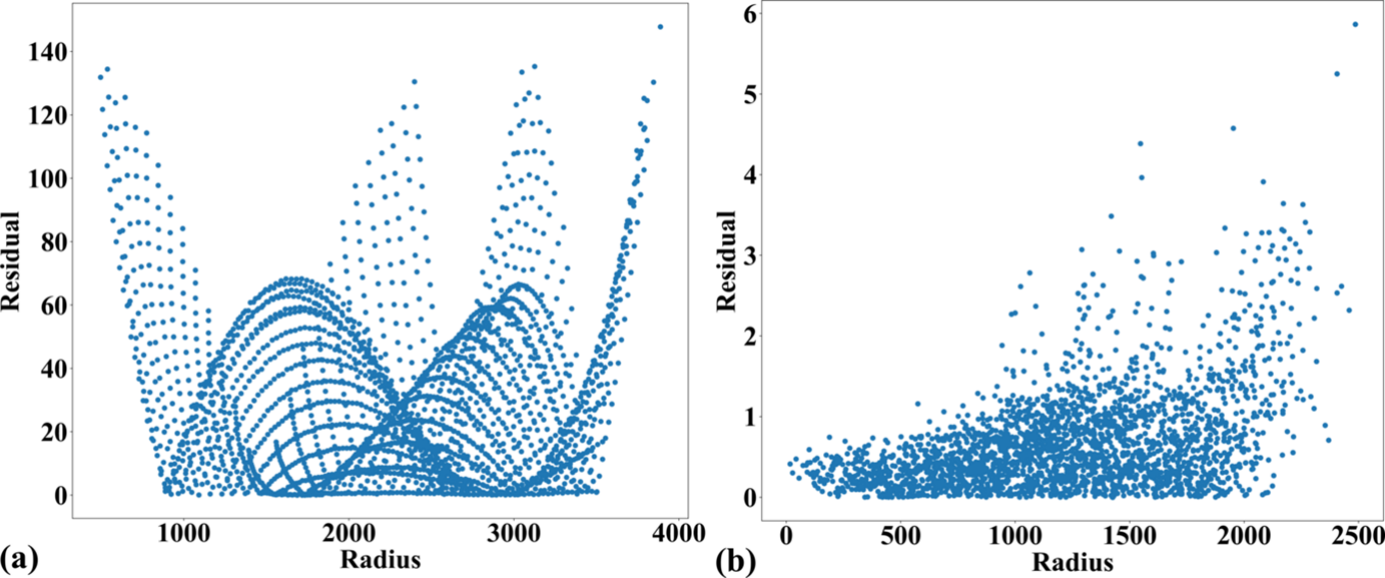
Plot of point distances from their fitted straight line (horizontal direction) versus their distances from the origin: (*a*) before radial distortion correction, (*b*) after correction.

To demonstrate the capability of the proposed methods for correcting strong distortion, we tested them on a line-pattern image acquired using a commercial GoPro camera. This camera provides a wide-angle view but suffers from strong radial distortion, commonly known as fisheye distortion. Additionally, as the image was taken handheld, it suffers from strong perspective distortion. The acquired image is processed through several steps: background normalization, detecting points on lines, and grouping points line by line in the horizontal and vertical directions, as shown in Fig. 12 ▸(*b*). This demonstrates the advantage of the new approach for grouping points on strongly curved lines, as described in Section 2.2. The calibration follows the same routine as described above: determining the distortion center, calibrating and correcting perspective distortion, then calculating the polynomial coefficients of radial distortion. Figs. 17 ▸ and 18 ▸ show the results of the applied correction. Note that only radial distortion needs to be corrected. As shown in Fig. 18 ▸, the point distances to the fitted line within their group before and after correction confirm that the method achieves great results. It is important to note that this is a standard visual camera, not specifically designed for scientific purposes, so reducing distortion to below 6 pixels across a range of 4000 pixels is a significant achievement.

### 3.2. Impact of distortion correction on tomographic data

**Figure 19:**
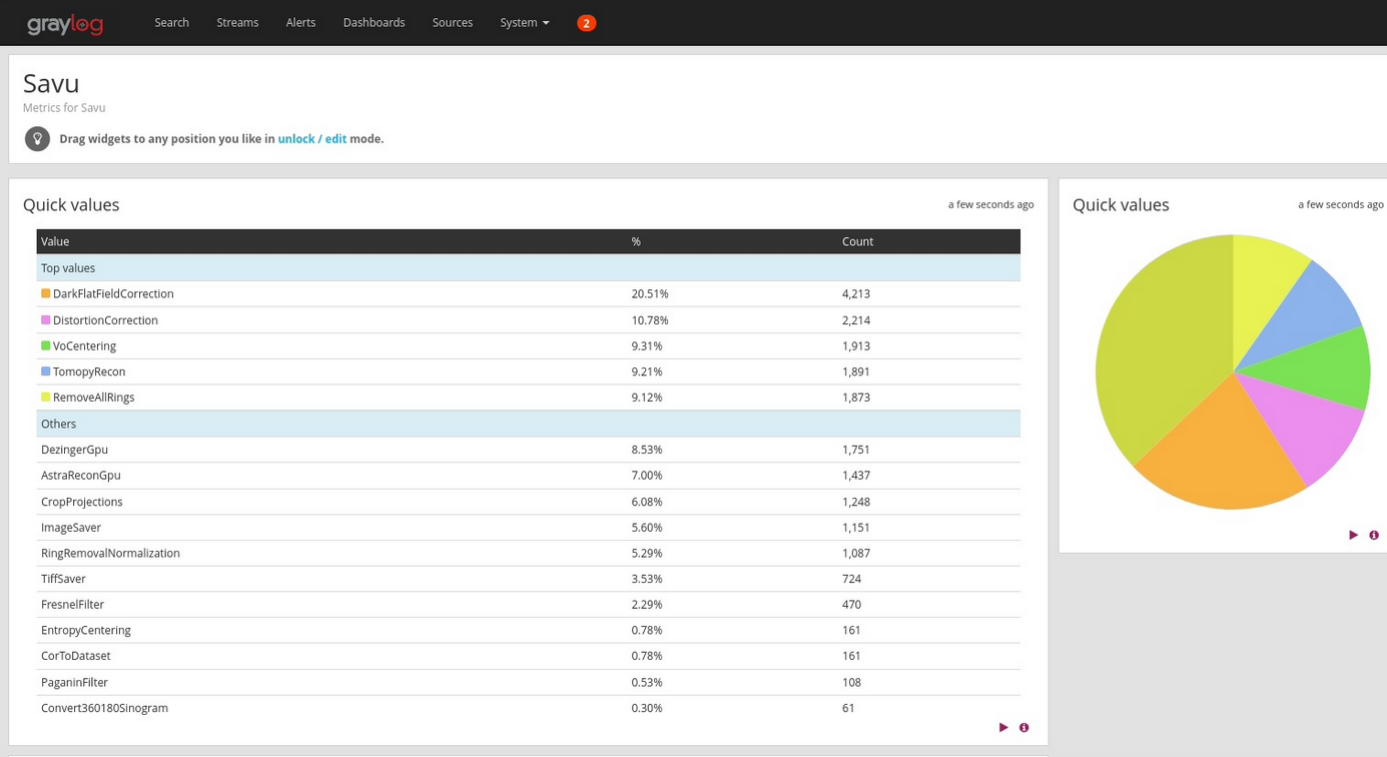
Statistical information on *Savu*’s plugin usage, as reported by the Graylog web service.

The primary application of *Discorpy* and its algorithms is to calibrate the radial distortion of lens-based detection systems used in synchrotron-based tomography. Beamline scientists use these detection systems to capture images of calibration targets, such as dot-pattern or line-pattern images. *Discorpy* is then used to calculate the distortion center and radial distortion coefficients of the system. These parameters are subsequently passed to tomographic software to undistort projections acquired with the same detector configuration, as explained in Section 2.6. This correction is implemented in several tomographic software packages, including *Tomopy* (Gürsoy *et al.*, 2014 ▸), *Savu* (Wadeson & Basham, 2016 ▸), and *Algotom* (Vo *et al.*, 2021 ▸). At beamlines I12 and I13 of Diamond Light Source, distortion measurement and correction are routinely performed as part of the standard workflow for tomographic data processing. Fig. 19 ▸ shows the statistical usage of data processing plugins in *Savu* software for tomographic workflows, as reported by the Graylog web service used for monitoring and analyzing plugin activity. The DistortionCorrection plugin is the second most popular. This highlights the significant role of distortion correction in the tomographic data processing workflow.

While distortion calibration and correction are routine at DLS, it is unclear why this step is not commonly performed at other tomography facilities, given the difficulty of achieving a distortion-free design for high-resolution lens-based detectors. One possible reason is the challenge of obtaining high-resolution calibration targets. At I13, this issue was addressed by fabricating a dot-target using lithography, provided by XRnanotech. Another alternative is to use a line-pattern target, which is easier to fabricate and commercially available from suppliers such as Thorlabs. The following demonstrates the importance of distortion correction in tomography and the need for efforts to ensure its implementation.

In a parallel-beam tomography system, acquired projections are reconstructed slice by slice, with each row of the projection images expected to be independent. However, radial distortion violates this condition, as it causes the sinogram to contain information from adjacent volumes moving in and out of a single row’s field of view. As a result, the reconstructed slice exhibits streak artifacts. The impact of distortion increases with radial distance from the optical axis, meaning artifacts become more strong toward the image borders. This pattern is characteristic of distortion-related artifacts and helps to distinguish them from streak artifacts caused by other problems, such as misalignment or an incorrect center of rotation. The streak artifacts and overlapping information between neighboring sinograms significantly impact tomographic applications where interface details are more critical than attenuation information, such as in analyzing crack formation (Cartwright-Taylor *et al.*, 2022 ▸), material porosity (Plachá *et al.*, 2024 ▸), or small structural features (*e.g.* grains, polymer fibers, *etc*.) (Chai *et al.*, 2020 ▸). Specifically, as shown by Strotton *et al.* (2018 ▸), users of the I13 beamline demonstrated in detail how distortion correction improves the accuracy of tomographic data for a rat spinal cord.

**Figure 20:**
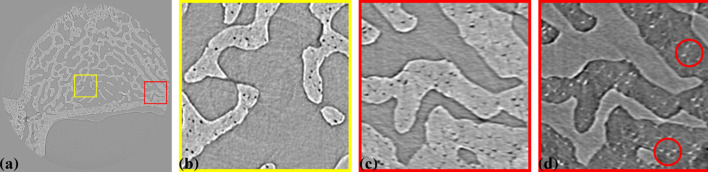
(*a*) Reconstructed slice of a bone sample without distortion correction. (*b*) Zoomed-in view of the yellow-framed area in (*a*). (*c*) Zoomed-in view of the red-framed area in (*a*). (*d*) Same as (*c*) but with inverted contrast.

Fig. 20 ▸ shows a reconstructed slice of tomography data without radial distortion correction, showing a cross-section of an intervertebral disk sample from a rat. This sample was used to study injury and degeneration of soft tissue under mechanical loading (Disney *et al.*, 2023 ▸). Here, the feature of interest is bone porosity. The detector configuration is the same as in Fig. 14 ▸, with 2001 projections and an X-ray energy of 27.6 keV. As seen in Fig. 20 ▸(*b*), a zoomed-in area in the middle of the slice [Fig. 20 ▸(*a*)], the streak artifacts are minimal, and the pores retain their shape. However, in Figs. 20 ▸(*c*) and 20 ▸(*d*) (with inverted contrast to enhance the pores), which focuses on an area near the edge of the slice, streak artifacts caused by radial distortion, despite being only 2.5 pixels [Fig. 16 ▸(*a*)], distort the shape of the pores. This distortion significantly impacts the analysis of pore distribution, shape, and volume.

**Figure 21:**
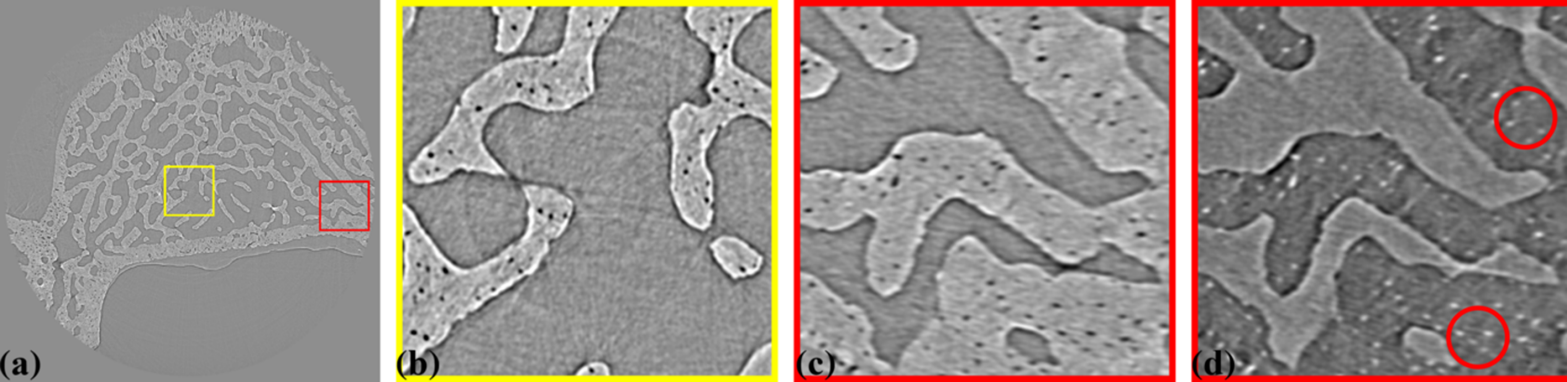
(*a*) Reconstructed slice of a bone sample with distortion correction. (*b*) Zoomed-in view of the yellow-framed area in (*a*). (*c*) Zoomed-in view of the red-framed area in (*a*). (*d*) Same as (*c*) but with inverted contrast.

Using the dot-pattern image shown in Fig. 14 ▸(*a*), the radial distortion coefficients and distortion center are determined, and the correction is applied to all projections of the tomographic data. For practical applications, a method is available in *Discorpy*, *Tomopy* and *Algotom* to generate an undistorted sinogram using neighboring sinograms, eliminating the need to process all projections or store intermediate data for subsequent processing steps. Fig. 21 ▸ shows the same sample slice after distortion correction. As seen, the shape of the pores in the previously distorted area is effectively restored.

**Figure 22:**
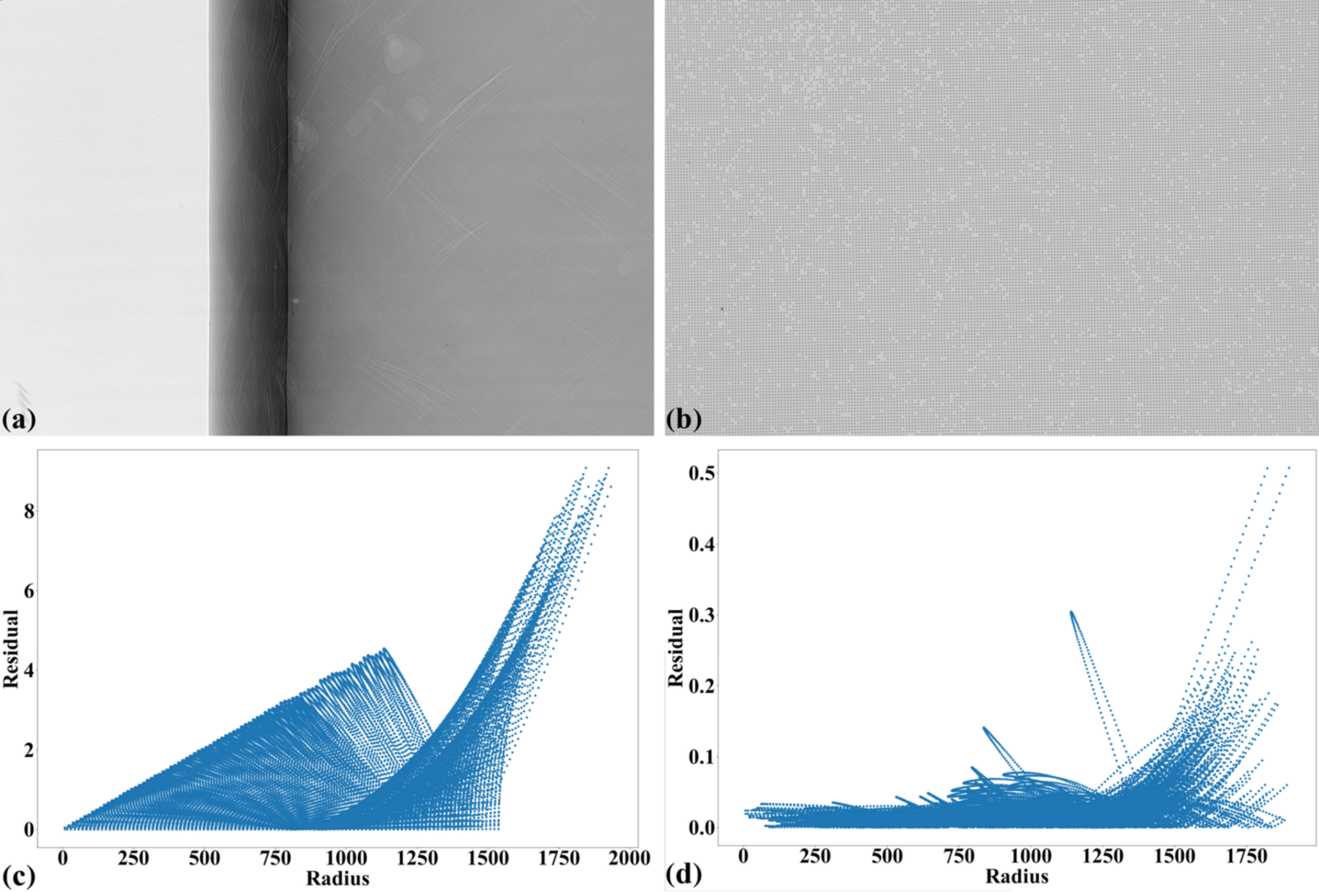
(*a*) Projection of a carbon-fiber tube with the rotation axis offset to the right side. (*b*) Dot pattern used to calculate distortion coefficients. (*c*) Plot of point distances from their fitted straight line in the horizontal direction versus their distances from the origin before radial distortion correction. (*d*) Same as (*c*) after correction.

Other tomographic techniques critically impacted by distortion are 360° offset rotation-axis scans, known as ‘half-acquisition’ scans for doubling the field of view, and grid scans. These methods require image stitching (Vo *et al.*, 2021 ▸), which becomes inaccurate or even impossible in the presence of distortion. Fig. 22 ▸(*a*) shows a projection from a tomographic dataset acquired using a half-acquisition scan to extend the field of view. The sample is a carbon-fiber reinforced polymer composite tube (Chai *et al.*, 2020 ▸), imaged at beamline I13 using a polychromatic ‘pink’ beam (20–24 keV) and a PCO4000 detector (4008 × 2672 pixels) with a pixel size of 2.25 µm. A total of 4501 projections were collected. Fig. 22 ▸(*b*) shows the dot pattern used to calculate the radial distortion coefficients and distortion center. The residual distortion before correction is approximately 9 pixels [Fig. 22 ▸(*c*)]. After correction, it is reduced to sub-pixel levels, as shown in Fig. 22 ▸(*d*).

**Figure 23:**
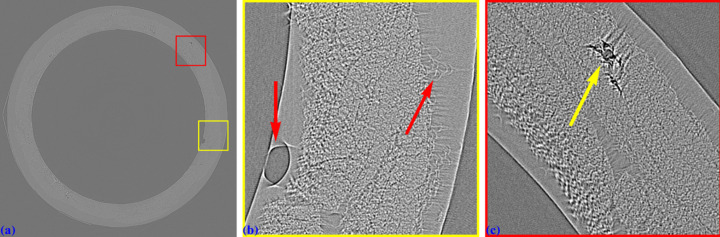
(*a*) Reconstructed slice at the middle row of the projection in Fig. 22 ▸(*a*). (*b*) Zoomed-in view of the yellow-framed area in (*a*). (*c*) Zoomed-in view of the red-framed area in (*a*).

Fig. 23 ▸(*a*) shows a reconstructed slice of the sample. The distortion introduces three major problems. First, the center of rotation cannot be determined precisely because it lies in the distorted region near the image border. The pixel size varies from the middle of the sinogram to the border. As a result, the two halves of the 360° sinogram cannot be properly stitched to form a 180° sinogram (Vo *et al.*, 2021 ▸). Even with manual alignment of the overlapping regions and visual inspection, no center of rotation produces an artifact-free slice. The second problem is that the rotation axis follows a curved path instead of a straight line perpendicular to the projection-image rows, meaning that the center of rotation must be determined for every sinogram. This is technically impractical. The third problem, as previously mentioned, is that distortion causes parts of the sample volume to shift in and out of the field of view of each sinogram, introducing streak artifacts. Note that streak artifacts caused by distortion can vary in direction, whereas those resulting from tomographic misalignment or an incorrect center of rotation are mainly oriented in the same direction. As seen in Figs. 23 ▸(*b*) and 23(*c*), the shape of the fibrous sample is distorted, and the damaged areas of the tube are also affected. Without distortion correction, it is impossible to accurately analyze the damage evolution of the sample under torsion testing (Chai *et al.*, 2020 ▸).

**Figure 24:**
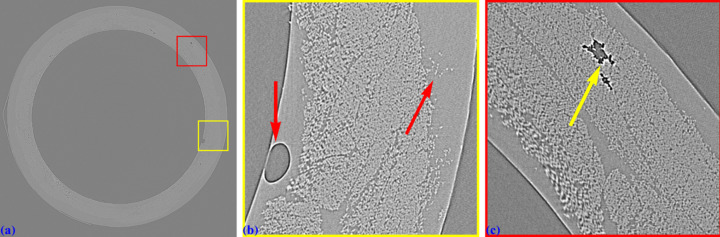
(*a*) Unwarped reconstructed slice at the same row used in Fig. 23 ▸(*a*). (*b*) Zoomed-in view of the yellow-framed area in (*a*). (*c*) Zoomed-in view of the red-framed area in (*a*).

Using the calculated distortion coefficients, an unwarped 360° sinogram at the same projection row is generated using nearby sinograms. The center of rotation or the overlap between the two halves of the sinogram is then determined using a method in the *Algotom* software, followed by slice reconstruction [Fig. 24 ▸(*a*)]. As seen, the cross-sectional shape of the fibers is restored, and the damaged areas are accurately recovered from distortion [Figs. 24 ▸(*b*) and 24 ▸(*c*)]. This ensures that the 3D analysis of the damage evolution of the sample under torsion provides reliable information.

## 4. Conclusions

This work presents methods for calibrating and correcting distortion in lens-based imaging systems, implemented in *Discorpy*. A key contribution is the ability to calibrate radial distortion even when perspective distortion and an offset optical center are present, using only a single calibration image. Unlike existing approaches that require multiple images or iterative optimization, the proposed method independently characterizes both distortion types with high accuracy, making *Discorpy* a practical tool for a wide range of imaging applications.

To accommodate different experimental conditions and enable a fully automated workflow, *Discorpy* supports various calibration patterns, including dot-pattern, line-pattern, and chessboard images. The software provides robust preprocessing methods for extracting reference points, handling image artifacts, and grouping points into lines.

Beyond calibration, this work highlights the critical role of distortion correction in tomography. Radial distortion introduces artifacts that affect reconstruction accuracy, particularly in applications requiring precise structural measurements. The results demonstrate how correction significantly improves image quality, preserving fine details. This is especially important for high-resolution synchrotron-based tomography, where sub-pixel accuracy is essential. *Discorpy* is open-source and designed for flexibility, allowing easy integration into existing workflows. With a modular implementation, detailed documentation, and a user-friendly API, it provides a reliable solution for researchers and engineers needing accurate distortion correction in imaging systems.

## Data Availability

The source code for *Discorpy* is available at: https://github.com/DiamondLightSource/discorpy or https://github.com/algotom/discorpy. Documentation for *Discorpy* can be found at: https://discorpy.readthedocs.io/. Various types of calibration images are accessible at: https://github.com/DiamondLightSource/discorpy/tree/master/data. The technical report on the initial development of *Discorpy* (formerly named Vounwarp) is available on the Zenodo platform: https://zenodo.org/records/1322720. Tomographic data demonstrating the impact of distortion correction is available at: https://zenodo.org/records/3339629.
